# Supplementary material for: Insights in Nutrition to Optimize Type 1 Diabetes Therapy
Source: Nutrients. 2024 Oct 26;16(21):3639. doi: 10.3390/nu16213639 (PMC11547730; doi:10.3390/nu16213639)
Supplement: Supplementary file 1 [file nutrients-16-03639-s001.zip › nutrients-3191950-supplementary.pdf]

Supplementary Materials

The overall choice of a correct diet in type 1 diabetes is essential for the therapeutic success, but as can be seen from the above-mentioned regimes of diet, there is not a univocal solution. Therefore, a series of different possibilities arise from the experience of the diabetologist, of the dietician and of the individual patient. While awaiting developments in research on nutrition in type 1 diabetes, it seems useful to add some personal considerations on how to approach nutrition and diet in this attachment.

1. Encourage food choices and refer to eating patterns that are normal for age and familiar tradition.

Personally, I have found it useful for children and adolescents to have an open discussion in groups, especially in summer camps or in meetings with patients, leaving a figurative menu available drawn up on the possibilities offered by the catering of the structure hosting the camp or meeting (Figure S1), in which the quantities of food and the corresponding glycemic load will be clearly represented with intelligible images even by small children. Of course, differences arise between patients, by first for those with celiac disease, to which the gluten free diet is mandatory.

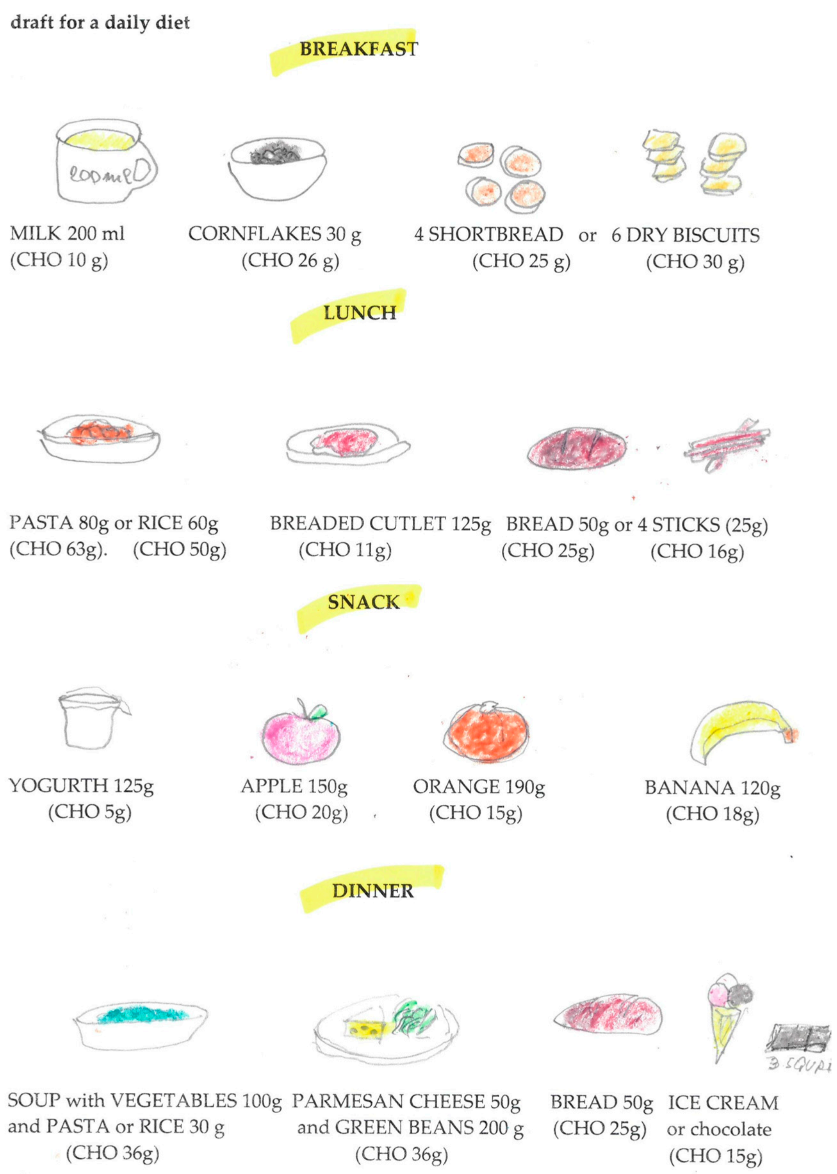

Figure S1. A draft for a daily diet.

Anyway, choosing is freedom for the patient, and eating in a facility with people without diabetes, sharing similar types and quantities of the same food, give to the diabetic ones a perception of an own 'near normality'.

2. Recognize the components of the meal, and be wary of unhealthy foods, because even if used by many, they are wrong for everyone.

Nutrition is changing rapidly under unfavorable marketing pressures. Given that always the superiority of the nutritional skills of type 1 diabetic people emerged compared to otherwise healthy subjects, it is the person with type 1 diabetes who should teach those without diabetes how to eat well.

3. Verify the impact of different foods on blood sugar levels after meals, and exploit the data detected by reflectometers or by glycemic sensors.

Identify the post-prandial time to peak, and verify its usefulness, to match insulin action with the post-meal glycemic peak. Teach each individual patient to anticipate a correction dose to the meal bolus dose, to improve insulin action of boluses.

4. Discuss within the group the most frequent situations that occur in everyday life: school, work, home, sport, play, holidays, parties, which in any case must all be addressed by adequate nutritional support. Unplanned physical activity is the most challenging test for patients with type 1 diabetes in real life. Regardless of insulin changes, provide ready-to-absorb carbohydrates, calibrated for body weight and duration of physical exercise, (0.5 g/kg weight per hour for adults and 1.0 g/kg weight per hour for youth) [102].
